# Splenic infarction during acute falciparum malaria: A case report

**DOI:** 10.3389/fmed.2022.951812

**Published:** 2022-09-14

**Authors:** Youguang Lu, Shian Zhang, Chuanshen Jiang

**Affiliations:** ^1^Department of Infectious Diseases, 900TH Hospital of Joint Logistics Support Force, Fuzhou, China; ^2^Department of Infectious Diseases, Fuzong Clinical College, Fujian Medical University, Fuzhou, China; ^3^Department of Hepatobiliary Disease, 900TH Hospital of Joint Logistics Support Force, Fuzhou, China; ^4^Department of Gastroenterology, 900TH Hospital of Joint Logistics Support Force, Fuzhou, China

**Keywords:** splenic infarction, malaria, *Plasmodium falciparum*, complication, treatment

## Abstract

Splenomegaly is common in malaria, but splenic infarction is a rare complication of malaria. We report a case of a patient with *Plasmodium falciparum* infection who developed abdominal pain, reappearance of fever, elevated D-dimer during treatment, and abdominal CT confirmed splenic infarction. The abdominal pain was relieved and the fever subsided by analgesic and anticoagulant therapy. Six months later, abdominal CT showed splenic recovery. As a result, splenic infarction should be considered when a patient with malaria developed abdominal pain, reappearance of fever and elevated blood D-dimer during treatment. In the absence of surgical indications, conservative medical treatment is effective.

## Introduction

Splenic infarction is a less common consequence of malaria that is likely underdiagnosed. We describe the case of a young guy who returned from Cameroon, Africa, with acute malaria and splenic infarction despite medical treatment and effective antimalarial treatment. The treatment was conservative, and the result was positive.

## Case report

On his return from Cameroon, Africa, a 36-year-old man with no previous exposure to malaria was admitted to the hospital with a fever. He had spent two months traveling across the country. He experienced a high-grade fever (39.8°C), chill, and headache two days after returning. Oral aspirin treatment showed no improvement after 2 days. He went to the hospital on the third day of his illness. Pallor, a fever of 39.3°C, and splenomegaly were discovered during the physical examination.

**FIGURE 1:**
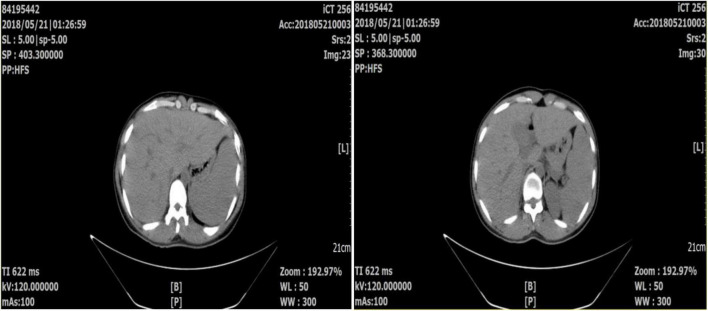
Abdominal computed tomography showed no splenic infarction.

**FIGURE 2:**
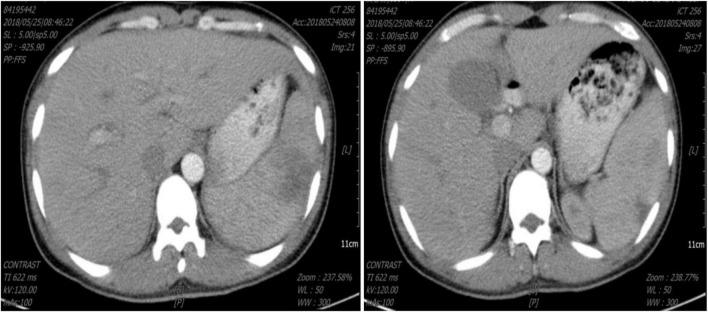
Abdominal computed tomography showed splenic infarction.

**FIGURE 3:**
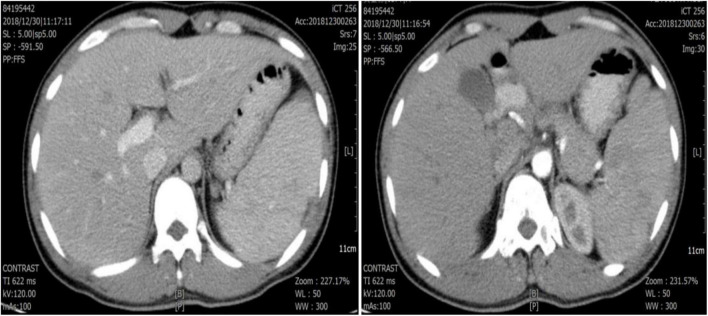
Abdominal computed tomography showed relief of splenic infarction.

A normal leukocyte count, hemoglobin (Hb) 8.6 g/dl, thrombocytopenia (platelet 32 × 10^9^/l), C-reactive protein (CRP) 45.5 mg/l, procalcitonin (PCT) 6.62 ng/ml, erythrocyte sedimentation (ESR) 93 mm/h, D-Dimer 1.58 mg/l and total/direct serum bilirubin of 34.2/24.5 μmol/L were discovered in the laboratory. Renal function and blood sugar levels were both within normal limits. The electrocardiogram (ECG) and chest radiography both came back normal. Abdominal computed tomography (CT) showed no hepatosplenomegaly (Figure 1). A blood smear investigation confirmed the *Plasmodium falciparum* diagnosis. Artesunate tablets were given to the patient, and the first dose was 100 mg orally, 50 mg once from the 2nd day, twice a day. For 2 days, the fever subsided. A full course (5 days) of artesunate tablets was continued. On the fourth day of treatment, the patient complained of pain in his left upper abdomen and a fever of 38.9°C. *Plasmodium falciparum* is not seen in peripheral blood smears. The spleen was bigger (5 cm) and sensitive on inspection. D-Dimer was found to be 5.24 mg/l in a lab test. Splenomegaly with several rounded, focused, low-density spots in various places (biggest 35 mm × 29 mm) suggested splenic infarct on abdominal CT (Figure 2). His hemodynamic condition was stable. For treatment, Nadroparin-calcium 0.4 mg was injected subcutaneously once a day for 5 days, and acetaminophen oxycodone tablets three times a day for pain relief. On day 3, after analgesic and anticoagulant treatment, abdominal pain was relieved, the fever subsided, and D-Dimer decreased (2.46 mg/l) on day 5. Thus emergent surgery was not required. Six months later, abdominal CT showed that the spleen infarction had recovered (Figure 3).

## Discussion

In China, imported malaria is common. Malaria is a parasitic infection caused by the malaria parasite, and its clinical manifestations include intermittent high fever, sweating, anemia, and spleen swelling. In 50–80% of malaria cases, the spleen is enlarged (1). The spleen is crucial in the host’s defense against *Plasmodium sp*. infection. Malaria can cause asymptomatic splenic enlargement and serious consequences such as splenic rupture, infarction, hyper-reactive malarial splenomegaly, hypersplenism, and splenic torsion (2). However, only a few occurrences of splenic infarction have been reported in malaria patients. Look for cases that have been recorded, most of which have been caused by *P. falciparum* infection (3). In patients with acute malaria, the spleen swelled mildly to moderately, which could be enlarged quickly. Hyperemia of the spleen, plasmodium infection, endotoxin, and allergic response are major possible causes (4). Because the splenic artery is terminal with few anastomotic branches, blood viscosity rises, and blood flow slows, making it easy for thrombosis to form for spleen infarction.

Lawrence analyzed 26 cases of splenic infarction in 2010 and found that the CT diagnostic rate was high, at 100% (5). In terms of finding infarcts and their range, CT has an edge. It can also rule out other disorders with similar clinical symptoms, like acute pancreatitis. As a result, the examination for peritoneal pain and fever, particularly left abdominal pain, might be improved to aid in the differential diagnosis.

Splenic infarction has no specific treatment; nonetheless, the outcome is usually favorable, and conservative management is the preferable option. A Pubmed search using the terms “malaria” and “splenic infarction” turned up several cases of splenic infarction associated with malaria, all of which were treated and survived (except one surgical treatment). Anticoagulation medication should be used to avoid infarction in other body sections. Patients with a substantial infarction region and splenic hematoma, splenic rupture, hemorrhagic shock, and splenic abscess should have their spleen surgically removed (6).

In this case, the patient was infected with *plasmodium falciparum* and developed spleen infarction, consistent with the statement that malaria with spleen infarction mostly occurred in *plasmodium falciparum* infection mentioned in the relevant literature. The diagnosis was confirmed by abdominal CT combined with elevated D-Dimer aggregates in blood. The symptoms of abdominal pain were relieved after analgesic and anticoagulant therapy without further progression to requiring surgical treatment, such as spleen rupture. It is suggested that analgesic and conservative anticoagulant therapy effectively treat malaria complicated with spleen infarction. To avoid further life-threatening complications, clinicians should consider the possibility of splenic infarction if a patient who received formal antimalarial treatment develops fever again, accompanied by left upper quadrant abdominal pain or enlarging tender splenomegaly during the treatment. Splenic infarction has no known early warning indications. Close medical monitoring, abdominal imaging (repeated if necessary), and detailed patient information are essential to ensure effective therapy of acute malaria and its complications.

In this case, no hepatosplenomegaly or splenic infarction was confirmed by abdominal CT examination before anti-plasmodium treatment. However, spleen infarction occurred 4 days after anti-plasmodium treatment. The adverse drug reactions of artemisinin and the case reports of malaria complicated with spleen infarction were reviewed, and there was no evidence of spleen infarction caused by artemisinin drugs. It is unclear whether the cause of spleen infarction is thrombosis of splenic blood vessels or the death of many *Plasmodium parasites* blocking splenic blood vessels, which needs to be further determined by similar pathologically confirmed cases.

## Data availability statement

The original contributions presented in this study are included in the article/supplementary material, further inquiries can be directed to the corresponding author.

## Ethics statement

Ethical review and approval was not required for the study on human participants in accordance with the local legislation and institutional requirements. The patients/participants provided their written informed consent to participate in this study.

## Author contributions

YL and CJ wrote the manuscript. CJ designed the manuscript. All authors approved the final version.
